# Clinical and genomic characterization of *Klebsiella pneumoniae* in a district hospital in Chengdu, China

**DOI:** 10.3389/fmicb.2025.1700934

**Published:** 2025-11-19

**Authors:** Yiyi Hu, Xingyu Lu, Yuyan Sun, Maowen Yu, Lei Zhang, Jianping Jiang, Juan Li

**Affiliations:** 1 Department of Infection Control, West China Second University Hospital, Sichuan University, Chengdu, China; 2 Department of Laboratory Medicine, Jintang First People’s Hospital, Chengdu, China; 3 Department of Medical Affairs, West China Hospital, Sichuan University, Chengdu, China; 4 Hospital Management Office, West China Hospital Sichuan University Jintang Hospital, Jintang First People’s Hospital, Chengdu, China; 5 Institute of Antibiotics, Huashan Hospital, Fudan University, Shanghai, China

**Keywords:** *Klebsiella pneumoniae*, resistance mechanisms, virulence, epidemiology, genomic analysis

## Abstract

*Klebsiella pneumoniae* is a significant human pathogen in both hospital and community settings; however, limited data exist regarding its prevalence in district-level hospitals. This study aimed to characterize the drug resistance mechanisms, molecular epidemiology, and virulence profiles of *K. pneumoniae* in a district hospital in Chengdu, China. A total of 114 clinical isolates were collected between May 2023 and May 2024. Antimicrobial susceptibility testing using the broth microdilution method revealed resistance rates of 14.0–21.1% to third-generation cephalosporins, and 5.3% to carbapenems. Whole-genome sequencing showed that 18.4% (21/114) of the isolates carried ESBL genes, with *bla*_CTX–M–15_ (*n* = 7) being the most common. Six carbapenem-resistant isolates were identified, of which four produced carbapenemases: three harbored *bla*_NDM–5_ and one carried *bla*_KPC–2_. MLST analysis identified 67 sequence types, with ST23 (*n* = 9) being the most prevalent. The 21 ESBL-producing isolates were distributed across 15 sequence types, while the six carbapenem-resistant isolates were assigned to four distinct sequence types. Virulence-associated genes were detected at high frequencies, including *ybt* (39.5%), *clb* (12.3%), *iuc* (50.0%), *iro* (60.5%), *rmpA* (59.6%), and *rmpA2* (36.8%), and were commonly found in ST23 (*n* = 9), ST412 (*n* = 6), ST25 (*n* = 5), ST268 (*n* = 5), and ST375 (*n* = 4). In conclusion, *K. pneumoniae* isolates from this district hospital showed low resistance rates but a worrying high prevalence of virulence genes. This highlights the urgent need for continuous surveillance and early intervention strategies to prevent the emergence of highly virulent and multidrug-resistant strains in healthcare settings.

## Introduction

*Klebsiella pneumoniae* is an important human pathogen both in hospital and community settings, which can cause a series of infections including respiratory tract infections, bloodstream infections, and urinary tract infections (Long et al., 2017; Martin and Bachman, 2018). Based on the data from the China Antimicrobial Surveillance Network (CHINET) in 2024, *K. pneumoniae* was the clinical isolate with the second highest detection rate, accounting for 13.9%. In the era of antibiotic resistance, *K. pneumoniae* has been an important multidrug-resistant (MDR) pathogen affecting humans worldwide (Navon-Venezia et al., 2017), and the extended-spectrum beta-lactamase (ESBL)-producing *K. pneumoniae* constitutes one of the most prevalent MDR bacterial pathogens worldwide (Breurec et al., 2013). Furthermore, increasing resistance to carbapenem in *K. pneumoniae* raises a global public health concern because of the prevalence of carbapenem-resistant *K. pneumoniae* (CRKP) and the associated high rate of mortality (Logan and Weinstein, 2017; Navon-Venezia et al., 2017).

Hypervirulent *K. pneumoniae* (hvKp) is another serious public health threat (Choby et al., 2020). Compared with classical *K. pneumoniae* (cKp), hvKp usually cause community-acquired infections, such as liver abscess and meningitis, with more severe clinical manifestations (Lee et al., 2006; Siu et al., 2012). Recently, multidrug-resistant hvKp (MDR-hvKp), mainly due to the acquisition of resistance genes by hvKp or the acquisition of virulence plasmids by cKp, has been reported globally (Hao et al., 2020; Shankar et al., 2020). For example, Jin et al. reported a hvKp strain acquired carbapenem resistance by obtaining a plasmid carrying *bla*_KPC–2_ (Jin et al., 2021), and Jiang et al. (2025) identified a novel IncFII_K34_ *bla*_KPC–2_ plasmid that drives the global emergence of carbapenem-resistant hvKp.

Current research on the epidemiology, antimicrobial resistance, and virulence factors of *K. pneumoniae* primarily focuses on large tertiary teaching hospitals, with less attention given to district-level hospitals (Hu et al., 2020; Lan et al., 2021). Furthermore, most studies have primarily focused on CRKP strains. Therefore, in this study, we investigated the molecular epidemiological characteristics, resistance mechanisms, and virulence profile of *K. pneumoniae* in a district-level hospital in Chengdu, China. These findings will provide an important basis for formulating effective measures to curb the rapid spread of MDR-Kp and hvKp isolates in district-level hospitals in China and other healthcare settings in developing countries and regions.

## Materials and methods

### Bacterial isolates

From May 2023 to May 2024, a total of 114 consecutive, non-duplicate clinical *K. pneumoniae* isolates were obtained from a district comprehensive tertiary hospital in Chengdu, China. All of the isolates were initially identified as *K. pneumoniae* using a Vitek II system (bioMerieux, Marcy-l’Etoile, France) according to the manufacturer’s recommendations. *K. pneumoniae* was grown on Luria-Bertani (LB) agar or broth at 37 °C. The isolates were stored at −80 °C in LB broth containing 30% glycerol (v/v) until used.

### Antimicrobial susceptibility testing

All of the clinical isolates were tested for susceptibility to 13 antimicrobial agents, including aztreonam, ceftazidime, cefotaxime, cefepime, cefoxitin, ciprofloxacin, gentamicin, amikacin, ertapenem, imipenem, meropenem, tigecycline, and piperacillin-tazobactam. Minimum inhibitory concentrations (MICs) of antimicrobial agents were determined using the broth microdilution method of the Clinical and Laboratory Standards Institute (CLSI, 2019 29th ed. Clinical and Laboratory Standards Institute; Wayne, PA: supplement M100.). The breakpoints of tigecycline were interpreted according to the US Food and Drug Administration (FDA). *E. coli* ATCC 25922 was used as a quality control strain. The definition of MDR was determined as non-susceptible to ≥1 agent in ≥3 antimicrobial categories according to an international expert proposal for interim standard definitions for acquired resistance (Magiorakos et al., 2012).

### Whole-genome sequencing and bioinformatics analysis

A total of 114 *K. pneumoniae* isolates were sequenced using a HiSeq X10 Sequencer (Illumina, San Diego, CA, USA), with 150 bp paired-end short reads and 200X coverage. Draft genomes were assembled using SPAdes v3.13.0 (Prjibelski et al., 2020). Kleborate v2.3 (Lam et al., 2021) was employed to screen the genome assemblies for the prediction of sequence type, capsular type and the detection of virulence loci, including hypermucoidy genes *rmpADC*, *rmpA2* and siderophore systems, such as yersiniabactin (*ybt*), colibactin (*clb*), aerobactin (*iuc*), and salmochelin (*iro*). Resistance genes were called by AMRFinderPlus v3.10.20 (Feldgarden et al., 2021). For phylogenetic analyses, core-genome alignments based on the concatenation of core genes were obtained using Panaroo v1.5.0 (Tonkin-Hill et al., 2020) with default parameters, where core genes were defined as those present in more than 98% of the genomes. Maximum-likelihood phylogenetic trees were constructed using FastTree v2.1.11 (model GTR + GAMMA) (Price et al., 2009). The tree was visualized using iTOL v6 (Letunic and Bork, 2024).

### Statistical analysis

Statistical significance was assessed using SPSS v.20.0 software (SPSS Inc., Chicago, IL, USA). Categorical variables, expressed as numbers and percentages, were compared by the Chi-square or Fisher’s exact test. The continuous data were expressed as mean ± standard deviation (mean ± SD) appropriately. *P-*values of <0.05 were considered to be significant.

## Results

### Clinical characteristics of *K. pneumoniae* isolates

Table 1 provides a summary of the clinical characteristics. A total of 114 isolates were mainly cultured from sputum (56.1%, 64/114), followed by urine (13.2%, 15/114) and pus (12.3%, 14/114). Most of the patients were male (64.0%, 73/114) with a mean age of 65.3 ± 17.3 years. Of the *K. pneumoniae* isolates, 36.8% (42/114) were obtained from the intensive care unit (ICU). These findings were consistent with previous studies that older adults are at increased risk of *K. pneumoniae* infection, and that the ICU remains a major hotspot for *K. pneumoniae* detection. More than half (50.9%, 58/114) of the patients have undergone invasive procedures, such as surgery, mechanical ventilation, or catheterization, which are closely related to the risk of *K. pneumoniae* infection. In addition, nearly half (46.5%, 53/114) of the patients have underlying comorbidities (e.g., diabetes, liver/kidney dysfunction), and six patients were in an immunocompromised state with HIV or cancer.

**TABLE 1:** Clinical characteristics of *K. pneumoniae* isolates.

Clinical characteristic	Value (*n* = 114)^#^
**Specimen**	
Sputum	64 (56.1)
Urine	15 (13.2)
Pus	14 (12.3)
Blood	12 (10.5)
Bronchoalveolar lavage fluid	4 (3.5)
Wound secretion	3 (2.6)
Drainage fluid	1 (0.9)
Tissue block	1 (0.9)
Age, years	65.3 ± 17.3
**Gender**	
Male	73 (64.0)
Female	41 (36.0)
**Clinical departments**	
Intensive care unit	42 (36.8)
Neurosurgery	12 (10.5)
Infectious disease	8 (7.0)
Gastrointestinal surgery	8 (7.0)
Urology surgery	7 (6.1)
Others	37 (32.5)
**Invasive procedures**	
Surgery	24 (21.1)
Mechanical ventilation	1 (0.9)
Catheterization	7 (6.1)
Multiple invasive procedures	26 (22.8)
None	56 (49.1)
**Comorbidities**	
Diabetes	13 (11.4)
Liver/kidney dysfunction	26 (22.8)
Multiple comorbidities	14 (12.3)
None	61 (53.5)
**Immune status**	
HIV	1 (0.9)
Cancer	5 (4.4)
Normal	108 (94.7)

^#^data was present in *n* (%).

### Antimicrobial susceptibility and antimicrobial resistance genes

**FIGURE 1:**
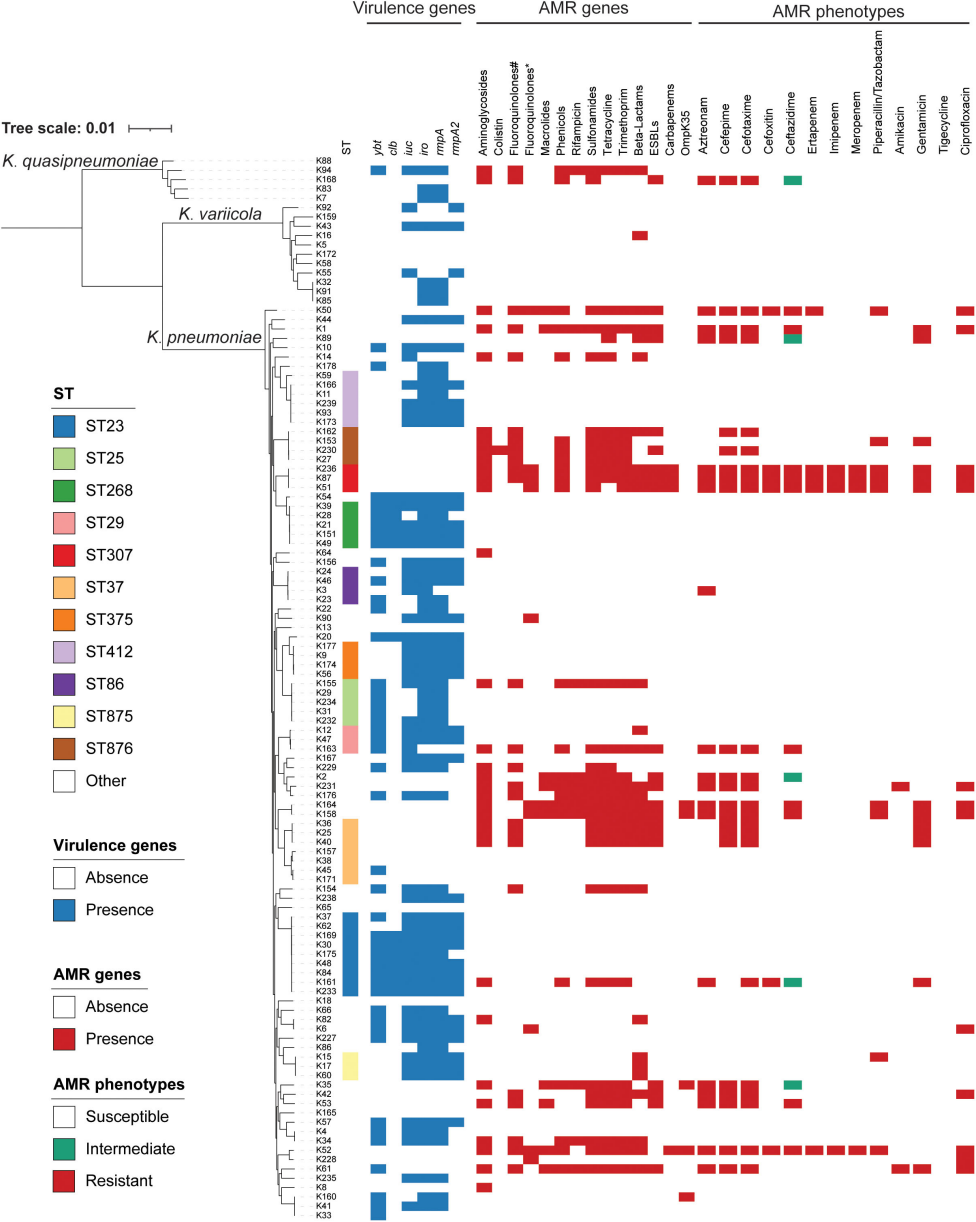
Phylogenetic analysis of 114 isolates. ST, virulence genes, AMR genes, and AMR phenotypes are indicated in different colors. The presence of OmpK35 stands for the mutation of OmpK35. ST, sequence typing; AMR, antimicrobial resistance; #fluoroquinolone resistance genes; *mutations found in the quinolone-resistance determining regions of GyrA and ParC.

Among 114 *K. pneumoniae* isolates, the prevalence of MDR isolates was 12.3% (14/114). As shown in Table 2, ESBL-producing isolates exhibited significantly higher resistance rates to 12 tested antimicrobial agents, including amikacin, aztreonam, cefotaxime, cefoxitin, ceftazidime, cefepime, ciprofloxacin, gentamicin, ertapenem, imipenem, meropenem, and piperacillin/tazobactam, compared to ESBL-negative isolates. The resistance rate to ceftazidime, cefotaxime, and cefepime were 14.0%, 21.1%, and 20.2%, respectively, which is consistent with the finding that 18.4% (21/114) of the *K. pneumoniae* isolates carried ESBL genes. Among *K. pneumoniae* isolates carrying ESBLs, the *bla*_CTX–M–15_ type (*n* = 7) was the most prevalent, followed by *bla*_CTX–M–14_ (*n* = 4) and *bla*_CTX–M–3_ (*n* = 4). Of concern, 5.3% (6/114) of the *K. pneumoniae* isolates were identified as carbapenem-resistant. Resistance to ertapenem was observed in all six isolates, with five also exhibiting non-susceptibility to both meropenem and imipenem. Four of the six carbapenem-resistant isolates were identified as carbapenemase producers, including three harboring *bla*_NDM–5_ and one carrying *bla*_KPC–2_. Additionally, the resistance rate to ciprofloxacin, amikacin, and gentamicin were 12.3% (14/114), 1.8% (2/114), and 11.4% (13/114), respectively, while fluoroquinolone and aminoglycoside resistance genes were detected in 21.9% (25/114) and 28.1% (32/114) of the isolates, respectively, with *qnrS1* (19/25) and *strA/B* (25/32) being the most prevalent. Notably, all *K. pneumoniae* isolates remained fully susceptible to tigecycline. The detailed phenotypic profile of antimicrobial resistance was shown in Figure 1, and the detailed genotypic profile of antimicrobial resistance was listed in Supplementary Table 1. Most of the OmpK35 were intact, and only five isolates (4.4%, 5/114) harbored truncated OmpK35 (Figure 1 and Supplementary Table 1).

**TABLE 2:** Antimicrobial susceptibility and carriage of resistance genes of *K. pneumoniae* isolates with or without ESBLs.

Antimicrobial agents	Total (*N* = 114)	ESBLs positive (*N* = 21)	ESBLs negative (*N* = 93)	*P*-value
Amikacin	2 (1.8)	2 (9.5)	0 (0.0)	**0.003**
Aztreonam	20 (17.5)	16 (76.2)	4 (4.3)	**<0.001**
Ceftazidime	16 (14.0)	13 (61.9)	3 (3.2)	**<0.001**
Cefotaxime	24 (21.1)	21 (100)	3 (3.2)	**<0.001**
Cefepime	23 (20.2)	21 (100)	2 (2.2)	**<0.001**
Cefoxitin	7 (6.1)	4 (19.0)	3 (3.2)	**0.006**
Ciprofloxacin	14 (12.3)	10 (47.6)	4 (4.3)	**<0.001**
Gentamicin	13 (11.4)	11 (52.4)	2 (2.2)	**<0.001**
Ertapenem	6 (5.3)	4 (19.0)	2 (2.2)	**0.002**
Imipenem	5 (4.4)	3 (14.3)	2 (2.2)	**0.014**
Meropenem	5 (4.4)	3 (14.3)	2 (2.2)	**0.014**
Tigecycline	0 (0.0)	0 (0.0)	0 (0.0)	**–**
Piperacillin /tazobactam	10 (8.8)	6 (28.6)	4 (4.3)	**<0.001**
**Resistance genes**				
ESBL	21 (18.4)	21 (100)	0 (0.0)	**<0.001**
CTX-M-15	7 (6.1)	7 (33.3)	0 (0.0)	**<0.001**
CTX-M-14	4 (3.5)	4 (19.0)	0 (0.0)	**<0.001**
CTX-M-3	4 (3.5)	4 (19.0)	0 (0.0)	**<0.001**
CTX-M-27	2 (1.8)	2 (9.5)	0 (0.0)	**0.003**
CTX-M-55	2 (1.8)	2 (9.5)	0 (0.0)	**0.003**
CTX-M-24	1 (0.9)	1 (4.8)	0 (0.0)	**0.032**
CTX-M-9	1 (0.9)	1 (4.8)	0 (0.0)	**0.032**
Carbapenemase	4 (3.5)	3 (14.3)	1 (1.1)	**0.003**
NDM-5	3 (2.6)	3 (14.3)	0 (0.0)	**<0.001**
KPC-2	1 (0.9)	0 (0.0)	1 (1.1)	0.638
Fluoroquinolone resistance	25 (21.9)	15 (71.4)	10 (10.8)	**<0.001**
Aminoglycoside resistance	32 (28.1)	19 (90.5)	13 (14.0)	**<0.001**

Data are number resistant (% of resistance rates). *P*-values are shown as ESBLs-positive isolates compared with ESBLs-negative isolates. Bold face indicates values that are significant (*P* < 0.05).

### Molecular epidemiology of *K. pneumoniae* isolates

The detailed molecular epidemiological characteristics of the *K. pneumoniae* isolates are presented in Figure 1. In this study, a total of 114 isolates belonging to the *K. pneumoniae* complex were identified, comprising three subspecies: *K. pneumoniae* (*n* = 98), *K. quasipneumoniae* subsp. *similipneumoniae* (*n* = 5), and *K. variicola* subsp. *variicola* (*n* = 11). MLST classified all isolates into 67 distinct sequence types (STs), with ST23 being the most prevalent (7.9%, 9/114), followed by ST37 (6.1%, 7/114) and ST412 (5.2%, 6/114). The 21 ESBL-positive *K. pneumoniae* isolates were distributed across 15 sequence types, with ST37 (*n* = 3) and ST307 (*n* = 3) being the most common, followed by ST876 (*n* = 2) and ST6839 (*n* = 2). The four carbapenemase-producing *K. pneumoniae* isolates belonged to two distinct sequence types: ST307, which harbored *bla*_NDM–5_ (*n* = 3), and ST709, which carried *bla*_KPC–2_ (*n* = 1).

### Capsular types and virulence-associated determinants

Among the 114 *K. pneumoniae* isolates, 67 were assigned to 19 distinct capsular (K) types, whereas the remaining 47 isolates were non-typeable. Among the 67 *K. pneumoniae* isolates with identifiable K-types, K2 was the most prevalent serotype (*n* = 17), followed by KL107 (*n* = 13) and K1 (*n* = 12). In addition, 91 of the 114 *K. pneumoniae* isolates were assigned to eight distinct O-serotypes, with O1ab being the most prevalent (*n* = 49), followed by O2a (*n* = 12) and O5 (*n* = 12); the remaining 23 isolates were O-non-typeable. Details of capsular and LPS serotypes were provided in Supplementary Table 1.

The distribution of virulence-associated genes among the isolates is also presented in Figure 1. Among the 114 clinical *K. pneumoniae* isolates, the prevalence of virulence-associated genes was as follows: *ybt* (39.5%, 45/114), *clb* (12.3%, 14/114), *iuc* (50.0%, 57/114), *iro* (60.5%, 69/114), *rmpA* (59.6%, 68/114), and *rmpA2* (36.8%, 42/114). In this study, the six virulence-associated genes were frequently detected in isolates belonging to ST23 (*n* = 9), ST412 (*n* = 6), ST25 (*n* = 5), ST268 (*n* = 5), and ST375 (*n* = 4), with ST23 being the most prevalent sequence type.

Although hypervirulent *K. pneumoniae* (HvKP) isolates are generally susceptible to most antimicrobial agents, the emergence of convergent *K. pneumoniae* strains that combine features of HvKP and CRKP has raised concern by linking virulence and resistance within single isolates (Chen and Kreiswirth, 2018). We did not identify such convergent isolates in our study; however, among *K. pneumoniae* isolates carrying the virulence-associated genes *ybt*, *clb*, *iuc*, *iro*, *rmpA*, and *rmpA2*, the proportions of MDR were 4.4% (2/45), 7.1% (1/14), 1.8% (1/57), 2.9% (2/69), 2.9% (2/68), and 2.4% (1/42), respectively. Details for all six virulence-associated genes are provided in Supplementary Table 1.

## Discussion

According to the 2024 report from the CHINET^1^, the resistance rate of *K. pneumoniae* to cefotaxime was 41.8%, and the prevalence of CRKP reached 22.6%. However, the prevalence of antimicrobial resistance varied across provinces in China. For example, in Sichuan province, the resistance rates to cefotaxime and carbapenems in 2023 were 26.3 and 9.0%, respectively, according to data from the China Antimicrobial Resistance Surveillance System (CARSS)^2^. Our findings revealed cefotaxime and carbapenem resistance rates of 21.1 and 5.3%, respectively, among *K. pneumoniae* isolates from a district-level tertiary hospital in Chengdu, Sichuan—both of which were lower than the corresponding averages reported for Sichuan Province.

CRKP is predominantly associated with ST11 in China and South America, whereas ST258 is the dominant lineage in Europe and the United States (Wang M. et al., 2022). ST11 is a single-locus variant of ST258, differing at the *tonB* gene, and both belong to clonal complex 258 (CC258) (Guo et al., 2022). In China, KPC-2 is the predominant carbapenemase among CRKP isolates, accounting for up to 94% of cases (Wang M. et al., 2022). However, in the present study, three of the four carbapenemase-producing *K. pneumoniae* isolates harbored *bla*_NDM–5_, while only one carried *bla*_KPC–2_. Notably, the three NDM-5-positive isolates belonged to ST307, whereas the KPC-2-producing isolate was assigned to ST709. ST307 is recognized as an emerging high-risk antimicrobial-resistant clone with global distribution and increasing prevalence; however, its occurrence in China has remained sporadic and infrequent. For example, a recent study reported an outbreak of ST307 CRKP isolates producing NDM-5 in a teaching hospital in Shanghai, China (Zhu et al., 2024).

In our study, two CRKP isolates did not harbor any carbapenemase genes. Notably, one of these isolates exhibited resistance to ertapenem alone, while remaining susceptible to meropenem and imipenem. A previous study demonstrated that mutations in *ramR* can lead to overexpression of efflux pumps and suppression of the outer membrane porin OmpK35, a mechanism specifically associated with ertapenem resistance in *K. pneumoniae* (Wang D. et al., 2022).

Notably, ST23—a prototypical lineage of hvKp—emerged as the most dominant sequence type among the isolates analyzed in this study. In addition, the prevalence of six virulence-associated genes—*ybt*, *clb*, *iuc*, *iro*, *rmpA*, and *rmpA2*—was relatively high, with three of them (*iuc*, *iro*, and *rmpA*) present in over 50% of the 114 *K. pneumoniae* isolates. A previous study reported that 37.8% *K. pneumoniae* isolates in China were hvKp defined by aerobactin detection, and the prevalence of hvKp varied among different cities, with the highest rate in Wuhan (73.9%) and the lowest in Zhejiang (8.3%) (Zhang et al., 2016). All six virulence-associated genes were most frequently detected in ST23, followed by ST25, ST268, ST375, and ST412. These sequence types have all been associated with high virulence. For example, previous studies have reported that ST25 *K. pneumoniae* strains are implicated in hospital-acquired infections (Cienfuegos-Gallet et al., 2019). Moreover, ST25 has also been identified as the predominant sequence type among carbapenem-resistant hvKp strains in a hospital in south-central China (Li et al., 2019). Multiple studies have indicated that ESBL-producing *K. pneumoniae* ST268 strains are strongly associated with hvKp (Ku et al., 2017; Falgenhauer et al., 2019; Kakuta et al., 2020). *K. pneumoniae* K2-ST375 is considered one of the most clinically concerning hvKp lineages, capable of causing pyogenic liver abscesses and primary lung abscesses (Hoashi et al., 2019; Hirai et al., 2020; Xie et al., 2021); A previous study of global ST412 *K. pneumoniae* strains revealed that the earliest strain lacked virulence plasmids; however, within approximately 2 years, they progressively acquired plasmids harboring virulence genes, leading to their evolution into hvKp (Liu et al., 2023). Nonetheless, further studies are needed to assess the virulence and fitness of the isolates characterized in this study.

## Limitation

In this study, the *K. pneumoniae* isolates were obtained from a single regional hospital, which may limit representativeness and generalizability. Although we compared our resistance rates with CHINET and CARSS (Qin et al., 2024) in the discussion, expanding the sample to include hospitals of different tiers would help clarify regional differences in resistance and virulence characteristics in future work.

## Conclusion

In summary, our study of *K. pneumoniae* isolates from a district-level hospital in Chengdu revealed slightly lower resistance rates to cefotaxime and carbapenems compared to the provincial averages in Sichuan. However, a high prevalence of virulence-associated genotypes was observed, warranting further investigation to demonstrate the virulent phenotypes and the pathogenicity. Although the prevalence of MDR-HvKP was extremely low in this study, our findings highlight the urgent need for continuous surveillance and early intervention strategies to prevent the emergence of highly virulent and multidrug-resistant isolates in healthcare settings.

## Data Availability

The genome sequences have been deposited in the DDBJ/ENA/GenBank under the bioproject PRJNA676183.

## Footnotes

1
https://www.chinets.com

2
https://www.carss.cn
